# Genomic Sequencing Reveals the Diversity of Seminal Bacteria and Relationships to Reproductive Potential in Boar Sperm

**DOI:** 10.3389/fmicb.2020.01873

**Published:** 2020-08-04

**Authors:** Jing Zhang, Huan Liu, Qiangzhen Yang, Peifei Li, Yi Wen, Xuejun Han, Bushe Li, Hongju Jiang, Xinhong Li

**Affiliations:** ^1^Shanghai Key Lab of Veterinary Biotechnology, School of Agriculture and Biology, Shanghai Jiao Tong University, Shanghai, China; ^2^Shanghai Engineering Research Center of Breeding Pig, Shanghai, China

**Keywords:** semen microbiota, seasonal variation, reproductive potential, sperm, boar

## Abstract

A number of emerging studies suggest that pathogenic microorganisms in semen may cause a decline in the reproductive potential of spermatozoa, and the bacterial diversity and profile of ejaculated boar semen in different seasons are currently unknown. To explore the bacterial composition and changes in ejaculated boar semen from winter and summer, and the underlying mechanism of decline in sperm quality and fertility capacity in summer, 120 ejaculated semen samples were examined for bacterial communities using genomic sequencing technology, and the associations between microbial composition and sperm reproductive potential were investigated. The results showed that Proteobacteria (57.53%), Firmicutes (31.17%), Bacteroidetes (4.24%), and Actinobacteria (3.41%) are the dominant phyla in the ejaculated semen, and the dominant genera were *Pseudomonas* (34.41%) and *Lactobacillus* (19.93%), which belong to the phyla of Proteobacteria and Firmicutes, respectively. Interestingly, the higher diversity of bacteria in ejaculated semen of winter differs from that of summer semen, potentially due to seasonal changes related to changes in semen quality and sperm fertilizing capacity. Furthermore, the highly abundant *Lactobacillus* in winter samples were positively associated with sperm quality and reproductive performance obtained from sows inseminated with such semen samples, while in contrast, the highly abundant *Pseudomonas* in summer samples was negatively associated with sperm quality and reproductive potential. Additionally, our results strongly indicated that *Lactobacillus* is not only a potential probiotic for semen quality and fertility potential but also beneficial for restraining the negative influence of *Pseudomonas*. Overall, our findings significantly contribute to the current understanding of the phenotypes and etiology of male “summer infertility,” and may represent a frontier in male reproductive disorders and possible early prevention against pathogenic bacteria.

## Introduction

Artificial insemination (AI) has shown tremendous growth worldwide in its application in the commercial swine industry (Riesenbeck, 2011), and semen quality is the most important indicator that determines the success of fertilization. Boar semen is the appropriate medium for the survival and proliferation of different kinds of bacteria (Maroto Martin et al., 2010). Previous studies have confirmed that the boar preputial diverticulum, external genitalia, systemic and/or urogenital tract infections, skin or fur may result in bacterial contamination of the semen (Althouse and Lu, 2005; Goldberg et al., 2013). Additionally, the risk of bacterial contamination of the ejaculate is inherent to semen collection and processing (Althouse et al., 2000). Although certain bacteria transmitted through semen are considered pathogenic factors that cause various clinical diseases in sows, most studies so far have focused exclusively on the identification of bacterial species (Althouse et al., 2000; Althouse and Lu, 2005; Althouse, 2008; Ubeda et al., 2013), biological impact on sperm quality (Sepulveda et al., 2013, 2016; Pinart et al., 2017) and impact on fertilizing capacity (Maroto Martin et al., 2010; Prieto-Martinez et al., 2014) during sperm storage and subsequent reproductive performance. Despite a substantial list of bacteria have been recovered from boar semen, most are associated with the animals themselves (Kuster and Althouse, 2016), and the identification of microbial diversity specific to freshly ejaculated semen has been rarely reported.

The reproductive performance of boar depends on many factors, such as age, nutrition and season (Kunavongkrit et al., 2005). Seasonal variations have been considered to have key roles that affect the quantity and quality of semen, which is of considerable economic significance to the pig breeding industry (Sancho et al., 2004; Peltoniemi and Virolainen, 2006; Sarlos et al., 2011). Numerous studies have confirmed that season can affect a variety of quality characteristics of boar semen (Dziekonska et al., 2014; Fraser et al., 2016). Traditional researchers have primarily focused their attention on the basic quality parameters of semen affected by season (Zasiadczyk et al., 2015), whereas the variation of bacterial diversity in semen caused by seasonal change has been neglected. The topic of infections and infertility is extremely interesting, and there are still many aspects to be covered. In particular, it is not known whether seasonal changes affect the contribution of pathogenic bacteria in semen, thereby affect sperm quality and reproductive potential. Despite a possible link between bacteria and semen quality, few studies have attempted to characterize the variation of seminal microbiota in different season in pig feeding.

Additionally, the most common bacterial identification methods performed in previous studies were based on culture methods or PCR (Bussalleu et al., 2011). The majority of studies only focused on a few types of bacteria and depended on qualitative analysis to discover correlations between semen microbiome and semen quality (Althouse et al., 2000; Althouse and Lu, 2005; Maroto Martin et al., 2010). To date, a comprehensive understanding of bacterial communities in livestock semen is still lacking. Recently, the development of genomic sequencing technology has allowed for intensive study of the microbial genome, and revealed that human and animal semen microbiota play important roles in the physiology and immunity of spermatozoa (Weng et al., 2014; Alfano et al., 2018; Chen et al., 2018; Baud et al., 2019; Mandar et al., 2019). The potential interaction between the semen microbiota and spermatozoa may influence the reproductive function and health of males and result in the developmental origins of adult health. For instance, inflammatory prostatitis has been shown to be associated with abundant microbial communities in semen (Mandar, 2013; Korrovits et al., 2017). We therefore used the boar as a model organism and performed high-throughput sequencing of 16S ribosomal RNA genes to compare the bacterial composition in semen in summer and winter, and determined whether there was interaction between beneficial bacteria and pathogenic bacteria in semen during preservation. In this study, we addressed the followings: (1) investigating the bacterial composition in ejaculated semen collected in summer and winter to determine whether bacterial communities can be influenced by seasonal variation; (2) detecting changes of infertility-related phenotypes in certain genetic backgrounds; (3) determining whether there is interaction between beneficial bacteria and pathogenic bacteria in sperm, as well as the inhibition of beneficial bacteria on the toxicity of pathogenic bacteria in sperm; (4) exploring relationships of seminal microbiota to reproductive potential of boar sperm. We aimed to reveal microbial diversity in freshly ejaculated semen and the possible impact of the microbiota on boar sperm quality and sow fertilizing capacity, which may be useful for developing novel biomarkers as well as for the diagnosis of reproductive disorders in livestock.

## Materials and Methods

### Study Design and Animal Sampling

A total of 120 healthy boars (1–2 years of age) were randomly selected from Shanghai Sunsing Livestock Co., Ltd., China. All experimental boars were exposed to the same rearing conditions, and the same genetic system was present in each breed. Experiments complied with the standards of institutional guidelines for ethics in animal experimentation (Rule number 86/609/EEC-24/11/86), and all experimental procedures were permitted by the Animal Ethics Committee of Shanghai Jiao Tong University. All chemical products were obtained from Sigma Aldrich (St. Louis, MO, United States) unless otherwise mentioned. All boars were submitted to a collection frequency of twice a week using the gloved-hand technique.

### Semen Collection and Processing

Sixty semen samples were collected from 20 Duroc, 20 Landrace and 20 Yorkshire boars by using the false mount method once a week in summer (August), and the remaining 60 semen samples were collected in winter (December). All boars were subject to a collection frequency of twice a week. High-throughput sequencing of the 16S ribosomal RNA gene was performed using a single sample approach. To minimize the contamination of semen bacteria in the process of collecting semen, semen collection and processing were performed according to the minimum bacterial contamination pattern recommended by Althouse et al. (2000). Before collecting semen, the floor was mopped with disinfectant. The reusable false female mount and room surface were sterilized by ultraviolet light, and personnel were protected from exposure using proper safety precautions. Vessels for collecting semen, including glassware, plastic ware and containers, were sterilized by multiple rinses in distilled water and then in 70% alcohol with sufficient ventilation for complete evaporation of residual alcohol. When collecting the semen, the false female mount, boar fur, preputial opening and surrounding area were sterilized with a single-use disposable wipe to minimize the contamination of the semen collection vessel with preputial fluid. Double gloves were used when trimming preputial fur around the preputial opening, and the outer glove was discarded after the preparation of the boar, allowing for a clean gloved hand for grasping the penis. Disposable vinyl gloves with hand disinfectant were employed to reduce the risk of cross-contamination between boars. The ejaculated semen was diverted from the sperm collection vessel to a sterile test tube for medical blood collection and then directly placed in liquid nitrogen for subsequent bacterial analysis.

### Semen Quality Measurement

One hundred twenty collected semen samples were examined for bacterial communities and seven fertilizing criteria for semen quality, including semen volume, sperm concentration, morphology, motility, plasma membrane and acrosomal integrity, and mitochondrial activity. The semen volume and abnormal sperm percentage were detected according to the conventional methods in our lab (Li et al., 2019). Sperm concentration was evaluated by optical density using a calibrated spectrophotometer (Shanghai Spectrophotometer Co., Ltd., Shanghai, China). Sperm motility was measured by a computer-assisted semen analysis (CASA) system (Hamilton Thorne Research, Beverly, MA, United States) (Navarrete et al., 2015). Sperm morphology was analyzed at 400X magnification using phase-contrast microscopy (Nikon Eclipse E600, Tokyo, Japan). Analysis was acquired from several fields containing at least 200 spermatozoa per sample, and sample analysis was based on the examination of 25 consecutive digitalized images. The acrosome integrity was detected after staining the sperm with fluorescein isothiocyanate-conjugated peanut agglutinin (PNA-FITC) as a marker for acrosomal status and propidium iodide (PI) as an indicator of live or dead sperm, and each sample was assessed before fluorescence analysis via flow cytometry (Beckman Coulter Ltd., Brea, CA, United States) (Santiani et al., 2016). The assessment of mitochondrial membrane potential used a JC-1-specific probe (Beyotime Institute of Biotechnology, Shanghai, China), and the observed fluorescent signals were recorded using flow cytometry. The FL-1 channel was used to detect JC-1 monomers, and the FL-2 channel was used to detect JC-1 aggregates. FL2/FL1 served as the value of Δψm.

### Preparation and Inoculation of Semen With *P. aeruginosa* and *L. casei*

After analyzing the sperm concentration, semen was added to each of the above-mentioned falcon tubes to achieve a final sperm concentration of 1 × 10^7^ spz/mL in a total volume of 20 mL. Each falcon tube was subsequently brought to the final volume of 20 mL with semen preservation reagent. Thus, the final infective concentrations of *Pseudomonas aeruginosa* (isolated strain from pig semen, temporarily named PA18) or *Lactobacillus casei* CGMCC 1.570 used in the study were 1 × 10^5^ cfu/mL (Bussalleu et al., 2011; Sepúlveda et al., 2014). The negative control tube contained 20 mL solution including semen at a final sperm concentration of 1 × 10^7^ spz/mL and preservation reagent. All tubes were stored at 17°C for 7 days.

### Sow Fertility Measuring

The reproductive index data of sows after artificial insemination using 120 semen samples were provided by Shanghai Sunsing livestock Co., Ltd.

### DNA Extraction and PCR Amplification

Total bacterial genomic DNA were extracted using Fast DNA SPIN extraction kits (MP Biomedicals, Santa Ana, CA, United States), following the manufacturer’s instructions, and stored at -20°C for further analysis. PCR amplification of the bacterial 16S rRNA gene V3–V4 region was performed using the forward primer 338F (5′-ACTCCTACGGGAGGCAGCA-3′) and the reverse primer 806R (5′-GGACTACHVGGGTWTCTAAT-3′). Sample-specific 7 bp barcodes were incorporated into the primers for multiplex sequencing. The PCR components contained 5 μL of Q5 reaction buffer (5×), 5 μL of Q5 High-Fidelity GC buffer (5×), 0.25 μL of Q5 High-Fidelity DNA Polymerase (5U/μL), 2 μl (2.5 mM) of dNTPs, 1 μL (10 μM) of each forward and reverse primer, 2 μL of DNA template, and 8.75 μL of ddH_2_O. The reaction conditions consisted of an initial 98°C for 2 min followed by 25 cycles of 98°C for 15 s, 55°C for 30 s, 72°C for 30 s, and a final extension of 72°C for 5 min. PCR amplicons were purified with Agencourt AMPure Beads (Beckman Coulter, Indianapolis, IN, United States) and quantified using the PicoGreen dsDNA Assay Kit (Invitrogen, Carlsbad, CA, United States). The final sequencing library was prepared by mixing the equal amount of purified PCR products, followed by end reparation with the addition of a poly (A) tail, and the amplicons were connected with each other with the sequencing adapters.

### MiSeq High-Throughput Sequencing and Analysis

Purified PCR products from the 120 samples were mixed with equal concentrations, and sequencing was performed using the Illumina MiSeq platform with the MiSeq Reagent Kit v3 at Shanghai Personal Biotechnology Co., Ltd. (Shanghai, China). Sequencing libraries were generated and analyzed according to previous studies (Yin et al., 2018).

The Quantitative Insights Into Microbial Ecology (QIIME, v1.8.0) pipeline was employed to process the sequencing data, as previously described (Caporaso et al., 2010). Briefly, raw sequencing reads with exact matches to the barcodes were assigned to respective samples and identified as valid sequences. The low-quality sequences were filtered through the following criteria (Gill et al., 2006; Chen and Jiang, 2014): sequences that had a length of < 150 bp, sequences that had average Phred scores of < 20, sequences that contained ambiguous bases, and sequences that contained mononucleotide repeats of > 8 bp. Paired-end reads were assembled using FLASH (Magoc and Salzberg, 2011). After chimera detection, the remaining high-quality sequences were clustered into operational taxonomic units (OTUs) at 97% sequence identity by UCLUST (Edgar, 2010). A representative sequence was selected from each OTU using default parameters. OTU taxonomic classification was conducted by BLAST searching the representative sequences set against the Greengenes Database (DeSantis et al., 2006) using the best hit (Altschul et al., 1997). An OTU table was further generated to record the abundance of each OTU in each sample and the taxonomy of these OTUs. OTUs containing less than 0.001% of total sequences across all samples were discarded. To minimize the difference in sequencing depth across samples, an averaged, rounded rarefied OTU table was generated by averaging 100 evenly resampled OTU subsets under 90% of the minimum sequencing depth for further analysis.

To investigate the diversity of the semen microbiota, alpha diversity analysis was made by using the OTU table. OTU-level alpha diversity indexes, such as Chao1 richness estimator (Chao, 1984) and Shannon diversity index were calculated. Sequence data analyses were mainly performed using the QIIME and R packages (v3.2.0). OTU-level ranked abundance curves were generated to compare the richness and evenness of OTUs among samples. Beta diversity analysis was performed to investigate the structural variation of microbial communities across samples using UniFrac distance metrics (Lozupone and Knight, 2005; Lozupone et al., 2007) and visualized via principal coordinate analysis (PCoA) (Ramette, 2007). Differences in the Unifrac distances for pairwise comparisons among groups were determined using Student’s *t*-test and the Monte Carlo permutation test with 1000 permutations and visualized through the box-and-whiskers plots. Principal component analysis (PCA) was also conducted based on the genus-level compositional profiles (Ramette, 2007). A Venn diagram was generated to visualize the shared and unique OTUs among samples or groups using the R package “Venn Diagram,” based on the occurrence of OTUs across samples/groups regardless of their relative abundance (Zaura et al., 2009). Taxon abundances at the phylum, class, order, family, genus and species levels were statistically compared among groups by Mann-Whitney U signed-rank test and Metastats (White et al., 2009). The function of the whole microbiota was predicted by PICRUSt (Phylogenetic investigation of communities by reconstruction of unobserved states), based on high-quality sequences (Langille et al., 2013). Spearman correlation coefficients were calculated for correlations between semen quality index (i.e., sperm motility and malformation rate), fertility performance (i.e., mean litter size and mean number of live offspring) and the presence of bacteria in the semen.

### Statistical Analysis

All data are indicated as the mean ± standard error of the mean (SEM), except microbial data. The variances were first analyzed using a homogeneity test. If the data met the assumption of homoscedasticity, the significance of differences in the means was determined by one-way analysis of variance (ANOVA) and subsequent Duncan’s multiple range test (SPSS 19.0 for Windows; SPSS Inc., Chicago, IL, United States). Probability values of less than or equal to 0.05 (*P* ≤ 0.05), 0.01 (*P* ≤ 0.01) were considered significant.

## Results

### Comparison of Boar Semen Quality Parameters and Fertility Potential Between Winter and Summer

**FIGURE 1:**
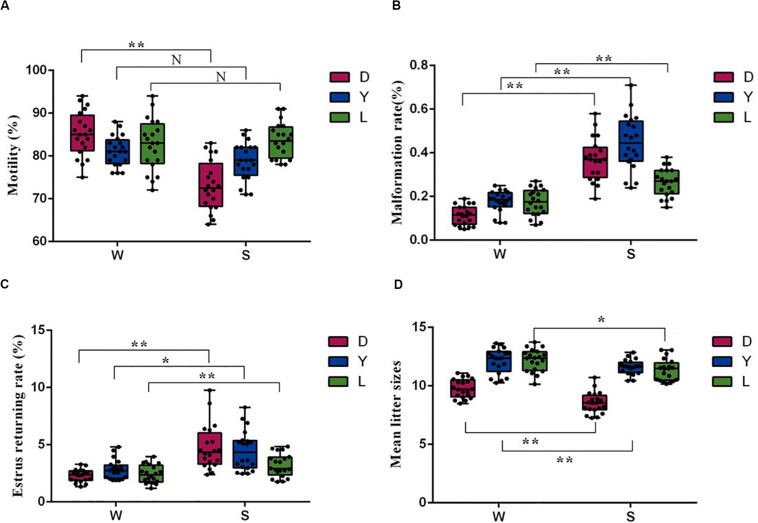
Comparison of sperm quality and reproductive parameters of sows after AI using 60 semen examples in winter (W) and in summer (S), respectively. **(A)** Sperm motility parameters, **(B)** Malformation rate, **(C)** Estrus returning rate (%), and **(D)** Mean litter size (*n* = 20, *P < 0.05, **P < 0.01). D, Duroc; L, Landrace; Y, Yorkshire.

Sperm motility and malformation rate are the fundamental indexes used to evaluate sperm fertility potential. To investigate whether the microbial community correlates with semen quality and reproductive potential, we first examined the difference in sperm quality between different seasons. As shown in Figures 1A,B, compared with winter, the Duroc semen samples collected in summer had significantly lower motility and higher sperm malformation (*P* < 0.01). There was no statistical difference in motility between Landrace and Yorkshire boars spermatozoa ejaculation in summer and winter (*P* > 0.05) (Figure 1A), and the Landrace and Yorkshire semen samples collected in summer had significantly higher sperm malformation compared with the winter samples (*P* < 0.01) (Figure 1B). Moreover, semen harvested in winter for artificial insemination had significantly lower estrus retuning rate in Duroc (*P* < 0.01), Yorkshire (*P* < 0.05) and Landrace (*P* < 0.01), whereas significantly higher mean litter size in Duroc (*P* < 0.01), Yorkshire (*P* < 0.01), and Landrace (*P* < 0.05) than that in summer (Figures 1C,D). There was no significant difference in quality parameters and fertility potential between different pig breeds in the same season. Given these data, we concluded that seasonal variations were the key factor affecting the quantity and quality of semen.

### Composition of Semen Microbiota in Boar

**FIGURE 2:**
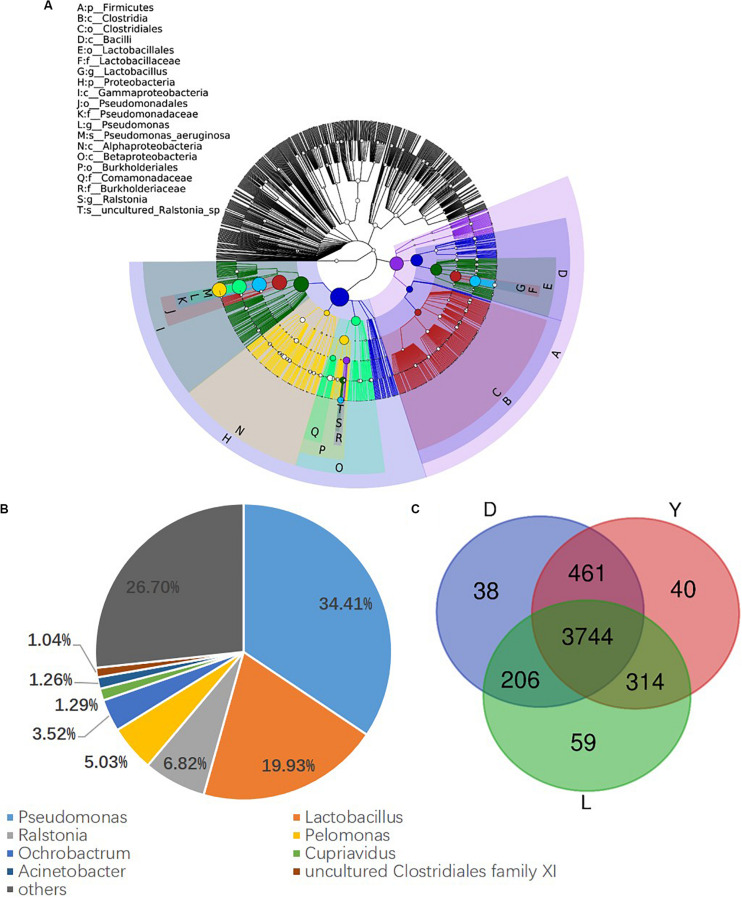
Phylogenetic tree of the taxa and bacterial composition of ejaculated semen. **(A)** Phylogenetic tree generated using GraPhlAn. The outermost circle represented phyla, and the inner circle represented genera. **(B)** Bacterial composition at genus level in semen. **(C)** Venn diagram of semen OTUs in different breed of boars. D, Duroc; L, Landrace; Y, Yorkshire.

**FIGURE 3:**
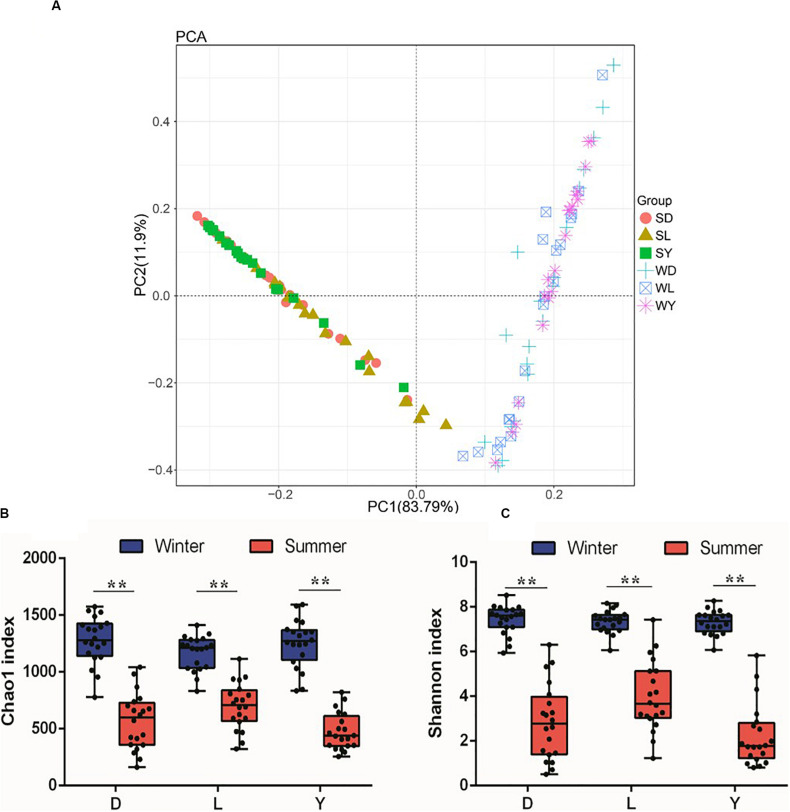
PCA analysis and diversity of semen microbiota in different breed of boars between winter and summer. **(A)** PCA plot. Each symbol represents 1 boar, SD, samples collected from Duroc in summer; SL, samples collected from Landrace in summer; SY, samples collected from Yorkshire in summer; WD, samples collected from Duroc in winter; WL, samples collected from Landrace in winter; WY, samples collected from Yorkshire in winter. **(B)** Chao1 richness index, and **(C)** Shannon diversity index. D, Duroc; L, Landrace; Y, Yorkshire. **P < 0.01.

To investigate the composition of ejaculated semen microbiota in boar, the microbial genomic DNA was isolated from 120 ejaculated boar semen samples. A total of 5450593 sequence reads was obtained from all samples, with an average read length 450 bp. Sequence reads were passed through our taxonomic mapping flow and classified to represent seminal bacteria. The dominant phyla were Proteobacteria (57.53 ± 30.04%), Firmicutes (31.17 ± 27.00%), Bacteroidetes (4.24 ± 3.82%), and Actinobacteria (3.41 ± 4.57%), with an average relative abundance of all samples higher than 1% (Figure 2A and Supplementary Figures 1, 2). The dominant microbial genera, with an average relative abundance of all samples higher than 1%, were *Pseudomonas* (34.41 ± 36.34%) and *Lactobacillus* (19.93 ± 25.91%), which belong to the phyla of Proteobacteria and Firmicutes, respectively (Figures 2A,B and Supplementary Figure 3). Breed specific OTUs were 38 in Duroc, 59 in Landrace, and 40 in Yorkshire, accompanied by 3744 common OTUs in these three breeds (Figure 2C). Principal Component Analysis revealed a separation of samples collected in winter and summer, and this obvious time-centered separation suggested a seasonal effect on the bacterial composition of semen, regardless of breed effect (Figure 3A). Furthermore, we calculated the microbial richness and diversity of samples based on the Chao 1 and Shannon indexes, respectively. Both indexes revealed that the microbial richness and diversity of samples collected in winter was significantly higher than that of samples collected in summer (Figures 3B,C) for all pig breeds. The higher Chao 1 and Shannon indexes of winter samples indicated higher richness and evenness of the bacteria community in semen in winter. Meanwhile, we observed a significant interaction between breeds and season. For summer samples, microbial richness (Chao 1) and diversity (Shannon index) were significantly higher in Landrace than Duroc and Yorkshire.

### Variance of Semen Microbiota in Different Seasons

**FIGURE 4:**
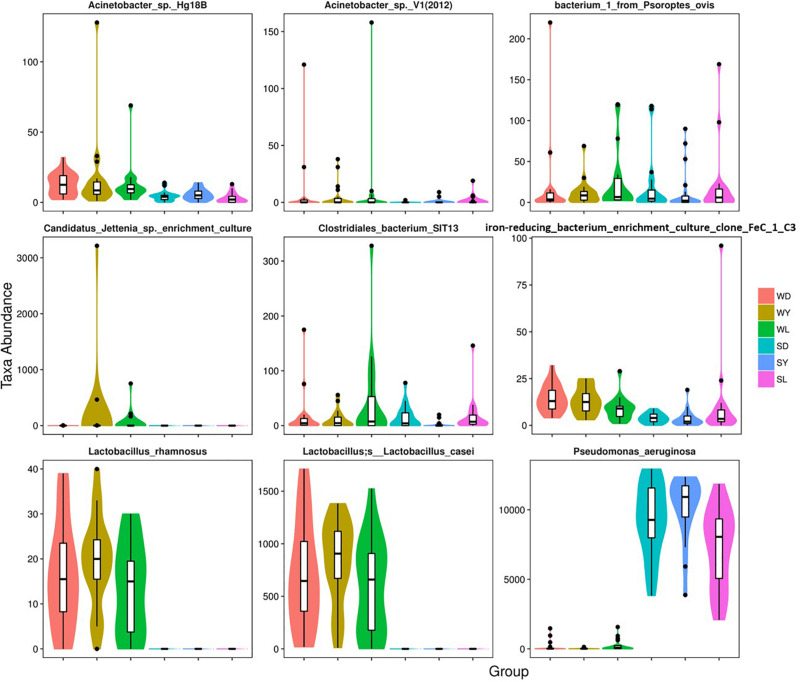
Dominant bacterial species with significantly different distributions between the winter and summer. SD, samples collected from Duroc in summer; SL, samples collected from Landrace in summer; SY, samples collected from Yorkshire in summer; WD, samples collected from Duroc in winter; WL, samples collected from Landrace in winter; WY, samples collected from Yorkshire in winter.

**FIGURE 5:**
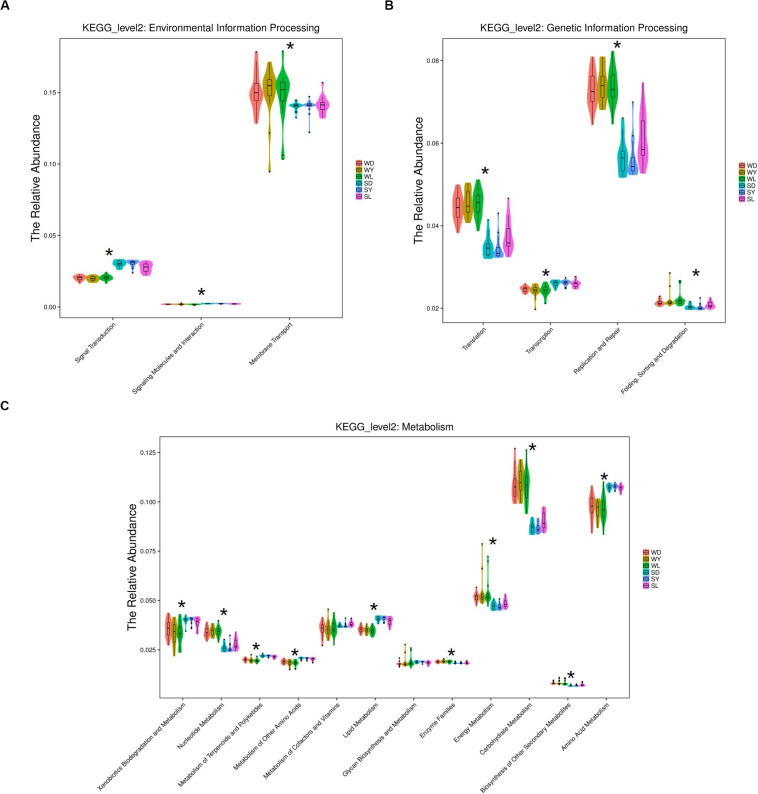
KEGG analysis and function prediction of semen bacteria in winter and summer. **(A)** Environmental information processing pathway, **(B)** genetic information processing pathway, and **(C)** metabolism pathway. WD, samples collected from Duroc in winter; WY, samples collected from Yorkshire in winter; WL, samples collected from Landrace in winter; SD, samples collected from Duroc in summer; SY, samples collected from Yorkshire in summer; SL, samples collected from Landrace in summer. The * means significant differences (*P* < 0.05) between summer and winter semen in all breeds of boars, and *P*-value was corrected for multiple testing according to the procedure of Benjamini-Hochberg.

To further investigate the seasonal effect on semen microbiota in boar, we compared the abundance of bacteria with significant difference between winter and summer. We observed a remarkable increasing of Proteobacteria in summer samples, whereas decreasing of Firmicutes, Bacteroidetes, Chloroflexi, and Verrucomicrobia at phylum level in all breeds (Table 1). With respect to genus level, there were 256, 238, and 75 bacterial groups significantly varied respectively in Duroc, Yorkshire, and Landrace summer samples compared to winter samples. In summer samples, *Pseudomonas* is significantly increased among all dominant genus, whereas *Lactobacillus*, *Pelomonas*, and *Prevotella_9* significantly decreased in all breeds (Table 2). Especially, the significant alternation of *Pseudomonas* and *Lactobacillus* mainly driven by a stimulation of *Pseudomonas aeruginosa*, as well as inhibition of *Lactobacillus rhamnosus* and *Lactobacillus casei* at species level (Figure 4). Moreover, functional prediction analysis of the whole microbiota by using PICRUSt revealed the relative abundance of 29 second level of functional categories of the KEGG pathway changed significantly between summer and winter in all breeds of boars (*P* < 0.05) (Supplementary Table 1). The active pathways (with an average relative abundance higher than 5%) of bacteria in winter semen are membrane transport (Figure 5A), replication and repair (Figure 5B), carbohydrate metabolism and energy metabolism (Figure 5C), whereas in summer semen were amino acid metabolism (Figure 5C) and an unclassified poorly characterized pathway (Supplementary Figure 4).

**TABLE 1:** Semen bacteria with different abundance at phylum level in winter and summer.

Phylum	Duroc				Landrace				Yorkshire			
	C.P^a^	Winter ARC^b^ (%)	Summer ARC^b^ (%)	Effect^c^	C.P^a^	Winter ARC^b^ (%)	Summer ARC^b^ (%)	Effect^c^	C.P^a^	Winter ARC^b^ (%)	Summer ARC^b^ (%)	Effect^c^
Acidobacteria	<0.01	0.94 ± 1.41	0.04 ± 0.06	–0.9	NS				<0.01	0.64 ± 0.48	0.04 ± 0.05	–0.6
Actinobacteria	NS				0.05	2.87 ± 5.48	5.77 ± 5.83	2.9	0.04	3.41 ± 4.7	1.93 ± 2.82	–1.48
Bacteroidetes	0.01	5.52 ± 4.41	2.63 ± 3.14	–2.89	0.02	6.50 ± 3.21	3.39 ± 2.64	–3.11	<0.01	4.83 ± 2.98	2.56 ± 4.67	–2.27
Chloroflexi	<0.01	0.88 ± 0.58	0.06 ± 0.06	–0.82	0.02	1.34 ± 3.14	0.21 ± 0.72	–1.13	<0.01	1.88 ± 3.67	0.07 ± 0.09	–1.81
Cyanobacteria	<0.01	0.16 ± 0.32	0.02 ± 0.02	–0.14	NS				<0.01	0.09 ± 0.06	0.03 ± 0.03	–0.06
Deinococcus-Thermus	<0.01	0.41 ± 0.28	0.15 ± 0.14	–0.26	NS				0.02	0.41 ± 0.33	0.15 ± 0.08	–0.26
Firmicutes	<0.01	52.35 ± 20.04	8.83 ± 8.25	–43.52	<0.01	51.74 ± 20.26	13.04 ± 11.05	–38.7	<0.01	56.38 ± 18.39	4.69 ± 5.26	–51.69
Fusobacteria	0.01	0.30 ± 0.68	0.04 ± 0.07	–0.26	NS				NS			
Gemmatimonadetes	<0.01	0.44 ± 0.32	0.01 ± 0.03	–0.43	NS				<0.01	0.40 ± 0.31	0.01 ± 0.01	–0.39
Ignavibacteriae	NS				0.04	0.86 ± 2.2	0.00 ± 0.00	–0.86	NS			
Planctomycetes	<0.01	0.05 ± 0.10	0.00 ± 0.01	–0.05	NS				0.02	2.03 ± 6.59	0.01 ± 0.02	–2.02
Proteobacteria	<0.01	34.17 ± 14.45	84.58 ± 13.28	50.41	<0.01	32.17 ± 16.62	76.12 ± 16	43.95	<0.01	27.93 ± 13.18	90.22 ± 11.35	62.29
Verrucomicrobia	<0.01	0.21 ± 0.12	0.03 ± 0.02	–0.18	<0.01	0.18 ± 0.15	0.04 ± 0.05	–0.14	<0.01	0.26 ± 0.23	0.03 ± 0.05	–0.23

*^a^C.P, P-value corrected for multiple testing according to the procedure of Benjamini-Hochberg. ^b^ARC, Average relative contribution (%) of a phylum. Values represent means ± SDs. The phyla with no significant variation or ARC lower than 0.1% in both winter and summer groups of all breeds of boars are not shown. ^c^Effect, indicates whether the average relative contribution of a phylum was increased (positive number) or decreased (negative number) in summer comparing to winter. NS, not significant.*

**TABLE 2:** Semen bacteria with different abundance at genus level in winter and summer.

Genus	Duroc				Landrace				Yorkshire			
	C.P^a^	Winter ARC^b^ (%)	Summer ARC^b^ (%)	Effect^c^	C.P^a^	Winter ARC^b^ (%)	Summer ARC^b^ (%)	Effect^c^	C.P^a^	Winter ARC^b^ (%)	Summer ARC^b^ (%)	Effect^c^
*Acinetobacter*	0.01	2.3 ± 6.54	0.41 ± 0.38	–1.89	NS				<0.01	1.05 ± 1.14	0.26 ± 0.12	–0.79
*Cupriavidus*	<0.01	2.66 ± 2.06	0.62 ± 0.68	–2.04	0.01	1.82 ± 1.94	0.58 ± 0.36	–1.24	NS			
*Lactobacillus*	<0.01	38.72 ± 26.35	0.54 ± 0.9	–38.18	<0.01	33.52 ± 24.52	0.77 ± 0.62	–32.75	<0.01	45.8 ± 21.13	0.2 ± 0.26	–45.6
*Ochrobactrum*	<0.01	4.69 ± 2.91	1.94 ± 1.35	–2.76	NS				0.01	4.36 ± 2.98	1.86 ± 1.64	–2.51
*Pelomonas*	<0.01	6.84 ± 2.88	3.38 ± 2.61	–3.46	0.01	7.19 ± 3.95	3.35 ± 1.95	–3.84	<0.01	6.57 ± 3.73	2.87 ± 1.92	–3.7
*Peptoclostridium*	0.02	0.77 ± 0.85	0.35 ± 0.45	–0.42	NS				<0.01	1.1 ± 1.15	0.15 ± 0.21	–0.95
*Prevotella_*9	<0.01	0.92 ± 0.69	0.24 ± 0.17	–0.68	<0.01	1.13 ± 0.86	0.06 ± 0.09	–1.08	<0.01	0.8 ± 0.84	0.22 ± 0.28	–0.58
*Pseudomonas*	<0.01	1.81 ± 3.36	70.33 ± 19.42	68.52	<0.01	2.14 ± 3.3	54.7 ± 21.2	52.56	<0.01	0.41 ± 0.31	77.07 ± 16.89	76.66
*Ralstonia*	<0.01	8.13 ± 3.38	4.25 ± 3.08	–3.88	NS				0.02	7.82 ± 4.33	4.46 ± 2.67	–3.36
Uncultured *Anaerolineaceae*	<0.01	0.23 ± 0.27	0.01 ± 0.02	–0.22	NS				<0.01	1.21 ± 3.33	0.01 ± 0.01	–1.21

*^a^C.P, P-value corrected for multiple testing according to the procedure of Benjamini-Hochberg. ^b^ARC, Average relative contribution (%) of a genus. Values represent means ± SDs. The genera with ARC lower than 1% in both winter and summer groups of all breeds of boars are not shown. ^c^Effect, indicates whether the average relative contribution of a phylum was increased (positive number) or decreased (negative number) in summer comparing to winter. NS, not significant.*

### Correlation Between Bacterial Genus and Semen Quality or Reproductive Performance

**FIGURE 6:**
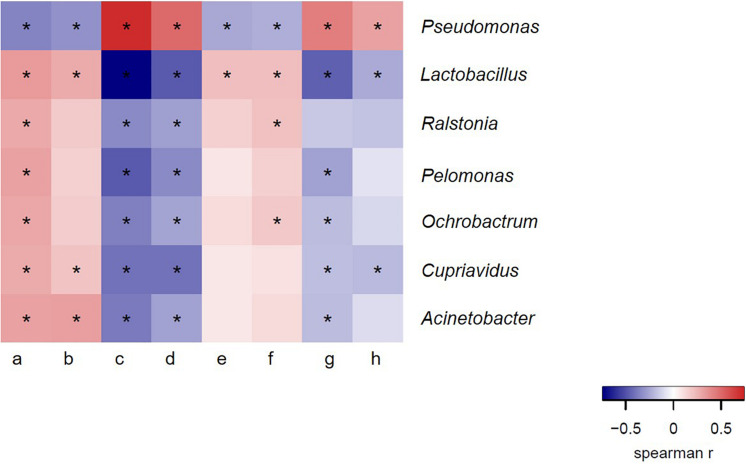
Spearman correlation coefficient between bacteria at genus level and semen quality, reproductive performances. a, sperm concentration; b, motility; c, sperm malformation rate; d, estrus returning rate; e, mean number of live offspring; f, mean litter size; g, mean number of stillbirth; h, mean number of weak offspring. The genera were sorted by their average relative contribution from high to low, and genera with the average relative contribution lower than 2% in both winter and summer groups or without significant variation of all breeds of boars are not shown. *Means significant correlation between bacteria and sperm reproductive potential (*P* < 0.05).

We therefore hypothesized that there was a certain correlation between bacteria in ejaculated semen, sperm quality and the reproductive performance obtained from sows inseminated with such semen samples. To verify this hypothesis, we next investigated whether there was a definite correlation between bacteria at genus level and sperm reproductive potential, and we carried out the correlation analysis between genus and sperm reproductive potential. Data from Spearman correlation coefficients showed that *Lactobacillus* had significantly positive (*P* < 0.05) correlations with sperm motility, mean litter size and mean number of live offspring of ejaculated semen, whereas significantly negative (*P* < 0.05) correlations with sperm malformation rate, estrus returning rate and mean number of stillbirths (Figure 6). In contrast, the presence of *Pseudomonas* in the ejaculate semen had significantly positive (*P* < 0.05) correlations with the sperm malformation rate, estrus returning rate and mean number of stillbirth, whereas significantly negative (*P* < 0.05) correlations with sperm motility, mean litter size and mean number of live offspring (Figure 6). These results revealed that there was a certain correlation between bacteria in ejaculated semen and sperm reproductive potential.

### Effects of Dominant Bacteria on Boar Sperm Motility Parameters for *in vitro* Preservation

**FIGURE 7:**
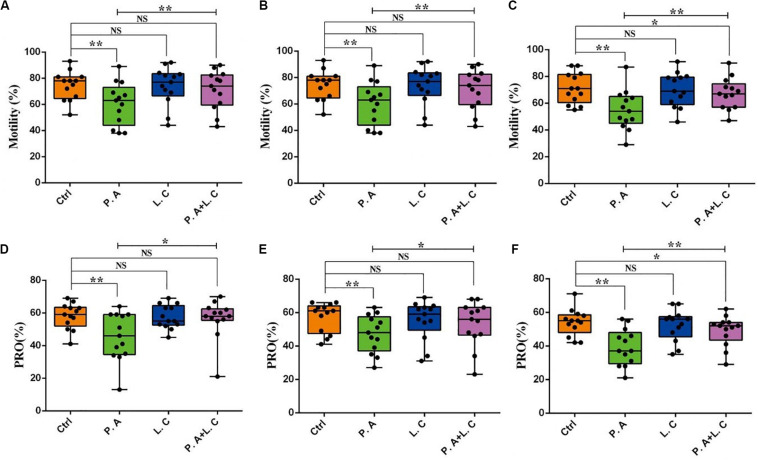
Dominant bacteria impact on motility parameters of boar sperm *in vitro* preservation. Total motility was determined on Day 3 **(A)**, Day 5 **(B)**, and Day 7 **(C)**, as well as progressive motility on Day 3 **(D)**, Day 5 **(E)**, and Day 7 **(F)**. Ctrl, control group; P.A, *Pseudomonas aeruginosa* treatment group; L.C, *Lactobacillus casei* treatment group; P.A + L.C, *Pseudomonas aeruginosa with Lactobacillus casei* treatment group. As mean ± SEM, the * means significant differences (*P* < 0.05), and the ** means highly significant differences (*P* < 0.01) between treatments at the same day; NS means no significant differences (*P* > 0.05). A total of 14 semen samples were used as biological replicates.

**FIGURE 8:**
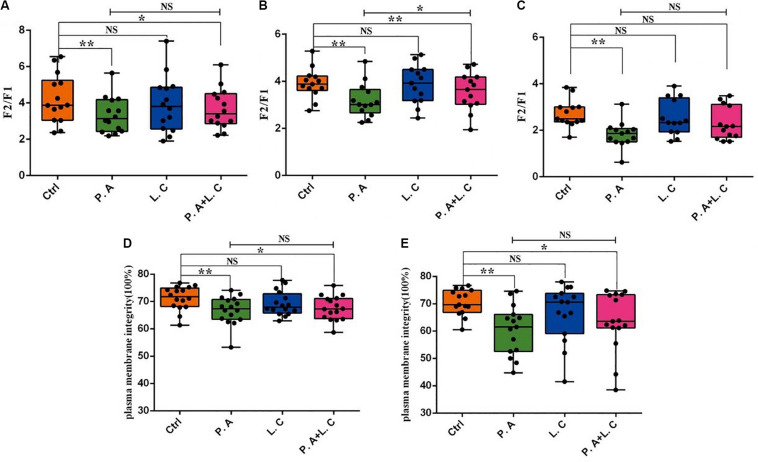
Dominant bacteria impact on boar sperm mitochondrial activity and plasma membrane integrity *in vitro* preservation. The mitochondrial activity was determined on Day 3 **(A)**, Day 5 **(B)**, and Day 7 **(C)**, as well as plasma membrane integrity on Day 5 **(D)** and Day 7 **(E)**. Ctrl, control group; P.A, *Pseudomonas aeruginosa* treatment group; L.C, *Lactobacillus casei* treatment group; P.A + L.C, *Pseudomonas aeruginosa with Lactobacillus casei* treatment group. As mean ± SEM, the * means significant differences (*P* < 0.05), and the ** means highly significant differences (*P* < 0.01) between treatments at the same day; NS means not significant differences (*P* > 0.05). A total of 14 semen samples were used as biological replicates.

To investigate whether there is a certain restraining effect between dominant beneficial bacteria and harmful bacteria, we evaluated the impact of adding two species of abundant bacteria (*Pseudomonas aeruginosa and Lactobacillus casei*) on the motility parameters, mitochondrial activity and plasma membrane integrity of sperm, and the inhibited ability of *Lactobacillus casei* to *Pseudomonas aeruginosa* which negatively affects the sperm quality of boar semen stored for 7 days. Our results showed that the total motility (MOT) and percentage of progressive motile spermatozoa (PRO) in the *P.A* + *L.C* treated group were significantly higher compared to the *P.A* treated group (*P* < 0.05), and were similar to the control group on days 3 and 5 (*P* > 0.05) (Figure 7). The *L.C* treated group did not show significant increases in the MOT and PRO compared to the control group (*P* > 0.05) (Figure 7). Furthermore, the results showed that *L. casei* significantly inhibited the damage of *P. aeruginosa* on the mitochondrial activity of sperm when stored *in vitro* on day 5 (Figure 8B). Although *L. casei* did not significantly inhibit the disruption of *P. aeruginosa* on the cytoplasmic membrane integrity of sperm on different days, all groups treated with *L. casei* showed a trend of inhibiting the damage of *P. aeruginosa* on the mitochondrial activity and cytoplasmic membrane integrity of sperm (Figure 8). The obtained results revealed that *Lactobacillus* is not only a potential probiotic for semen quality and fertility potential, but also may be beneficial in restraining the negative influence of *Pseudomonas* on sperm.

## Discussion

In the current study, microbial classifications revealed that a maximum of 24 phyla and 291 genera were present in semen samples, and our results more accurately and comprehensively reflected the diversity of microorganisms in ejaculated semen of boar breeding under certain conditions. Many factors affect the microbial community in ejaculated boar semen, including the process of semen collection. However, the collection of ejaculated semen and processing were performed according to the minimum bacterial patterns by Althouse et al. (2000). Therefore, the identified results of microbial diversity were authentic (creditable believable). Unexpectedly, the most abundant genera among all samples were *Pseudomonas* and *Lactobacillus*, and these identified results were different from the results of previous investigations (Althouse and Lu, 2005; Maroto Martin et al., 2010). Given the present results, we surmised that the dominant bacteria in boar semen may have diverse origins in different feeding environments and different feeding conditions.

It has been hypothesized that seasonal variation can influence the reproductive performance of pigs (Auvigne et al., 2010; De Rensis et al., 2017). However, whether seasonal variation can affect the distribution of dominant bacteria in livestock semen has not been reported. Our results revealed that there were significant differences in the bacterial community between winter and summer semen samples, and the relative abundance of the *Pseudomonas* genus was significantly increased compared to a reduction in the relative abundance of *Lactobacillus* genus in summer semen. In contrast, the proportion of *Lactobacillus* was significantly higher, while that of *Pseudomonas* was significantly lower in winter samples. Interestingly, there were significant differences in diversity of bacteria between winter and summer semen samples, and the higher species diversity in semen was observed in winter compared with that in summer semen samples. This finding is contrary to predictions that the diversity of bacteria is more abundant in burning hot summer. These conflicting findings may be due to the different microbial exposure of boars in the surrounding environment in winter and summer. Recently, lots of studies have indicated that many external factors can influence the colonization of human and animal microbiota. For example, there are many factors that influence the development of human and animal intestinal microbiota, such as feeding, antibiotic and probiotic treatment, as well as the microbial exposure. The exposure to microbes in the surrounding environment of human and animals is a strong influencing factor in the development of the intestinal microbiota. Different raising environments were shown to be associated with major differences in intestinal microbial diversity of pigs (Mulder et al., 2009). With respect to the pig raising environment, the stringency of the ultraviolet radiation that has a certain bactericidal effect and results in the decrease of bacteria in the pigpen in summer (Baubinas et al., 1983; Kraemer et al., 2018). On the other hand, the high temperature in summer strengthens disease prevention and control, especially using much more antibiotics to reduce the production of bacteria to some extent (Espigares et al., 2017; Johnson et al., 2017). Although there is no direct evidence linking microbial diversity in summer boar semen to antibiotic use, the high dosage utilization of antibiotics can alter the composition of gut microbes in pigs (Zhang et al., 2018). In addition, we speculated that the high abundance of *Pseudomonas* in semen in summer may inhibit the proliferation of other bacterial species. In the current study, we found that the seasonal changes dramatically altered the composition of boar semen bacteria, and the changes in microbial composition were related to the reproductive potential of spermatozoa and the fertility performance obtained from sows inseminated with such semen samples. Our study strongly revealed that the enrichment of seminal bacteria is associated with seasonal variation, expressed as an increase in diversity of seminal bacteria in winter compared to in summer, and supporting the hypothesis that the microbial colonizers harbored and the microbial diversity in semen were influenced by environmental factors.

Heat stress is recognized as the main factor of summer infertility inducing the decrease of reproductive potential of boar sperm (Maroto Martin et al., 2010; Kuster and Althouse, 2016). Some previous studies have identified bacteria in semen as a potential factor in male infertility. In several circumstances, male infertility has been linked to bacterial infections of the genital tract and infection of accessory sex gland (Gimenes et al., 2014), and the seminal bacteria community types were highly associated with semen quality, in particular. However, the true impact of bacterial infections on male fertility remains controversial. Pathogenic bacteria such as *Escherichia coli* (Bussalleu et al., 2011), *Enterobacteria* (Ubeda et al., 2013), *Clostridium perpringens* (Sepulveda et al., 2013), *Pseudomonas aeruginosa* (Sepúlveda et al., 2014), *Enterobacter cloacae* (Prieto-Martinez et al., 2014), and *Aeromonas hydrophila* (Boonthai et al., 2016) in semen were previously considered to be negatively associated with the sperm motility parameters, structural integrity, biochemical and physiological of sperm capacitation, even leading to suboptimal reproductive performance (Maroto Martin et al., 2010). However, the correlation between bacteria in freshly ejaculated boar semen and sperm reproductive potential indexes is not clear. In this study, the results indicated that the composition of dominant bacteria in boar semen changed greatly in winter and summer; *Pseudomonas aeruginosa* in summer ejaculated semen was the dominant bacterium, whereas *Lactobacillus* and other beneficial bacteria in winter semen became the dominant bacteria. Previous studies demonstrated that *Pseudomonas aeruginosa* had a great destructive effect on sperm quality characteristics and reproductive potential indicators during the *in vitro* preservation of boar semen (Sepulveda et al., 2016). Given these findings, we anticipated that the changes in microbial composition may be relate to the reproductive potential of spermatozoa. To examine whether there was a certain correlation between summer infertility and the semen pathogenic microorganisms, we investigated whole seminal bacterial communities and provided the most comprehensive analysis of the association between bacterial community and semen quality. The analysis results showed that seminal bacteria community types were highly associated with semen quality and the fertility performance obtained from sows inseminated with such semen samples. The high-abundance *Lactobacillus* in winter samples were positively associated with the motility of sperm, mean number of live offspring and mean litter size of sows and negatively associated with sperm malformation rate, estrus returning rate and mean number of stillbirths of sows. Nevertheless, the high-abundance *Pseudomonas* in summer samples was positively associated with sperm malformation rate, estrus returning rate and mean number of stillbirth of sows, while it was negatively associated with motility of sperm and mean litter size of sows. The present study suggested that summer sterility by *Pseudomonas* may occur because of reduced fertilizing capacity of the sperm and induction of a uterine environment hostile to sperm or embryonic survival (Arora et al., 2017). Taken together, these data confirmed that the quality of boar semen and reproductive potential of sows in summer are significantly affected by bacterial communities, and the seminal microbiome of summer samples with relatively lesser motility and abnormal sperm was explored, which may be useful for developing novel biomarkers as well as for diagnosis of reproductive disorders in livestock.

The intestinal microbiota is a complex micro-ecosystem, and there are certain mutual relationships among different bacteria. Under normal physiological conditions, intestinal microbiota is in a relatively stable state and then perform the physiological functions of different bacteria. It is not clear whether the dominant bacteria contained in boar semen also have similar characteristics to those of intestinal microbiota of animals, and whether there is a certain restraint effect between beneficial bacteria and potentially pathogenic bacteria. For bacteria in mammalian semen, traditional researchers always focused their attention on the destructive effects of potentially pathogenic bacteria such as *Pseudomonas* (Sepulveda et al., 2013, Sepúlveda et al., 2014), but often neglected the positive regulation effects of beneficial bacteria such as *Lactobacillus*. Recently, Barbonetti et al. suggested that *Lactobacillus* may be helpful in countering the negative influence of *Escherichia coli* on male sperm (Weng et al., 2014). To gain insight into the potential role of *Lactobacillus* for semen quality maintenance and test whether it is helpful in countering the negative influence of *Pseudomonas aeruginosa*, we investigated the interaction between *Pseudomonas aeruginosa* and *Lactobacillus casei* together with sperm *in vitro* preservation at 17°C. Our study revealed that *Lactobacillus casei* was a potential probiotic for maintaining semen quality and helpful in countering the negative influence of *Pseudomonas aeruginosa*. Meanwhile, the destructive inhibition of *Lactobacillus casei* on *Pseudomonas aeruginosa* disruption may provide a theoretical basis for us to effectively control pathogenic microorganisms in semen in summer. Furthermore, our investigation showed that boar semen in winter has a specific microbiota milieu, such as *Lactobacillus* genera, which may not be detrimental to spermatozoa but necessary for normal sperm physiological function (Hou et al., 2013; Weng et al., 2014; Mandar et al., 2017). Accordingly, we could conclude that there is a certain restraint effect between *Lactobacillus casei* and *Pseudomonas aeruginosa*, and the effect of contamination on sperm quality as well as sperm-bacteria interactions depends on the bacteria type. To our knowledge, this is the first exploration of the impact factors underlying seasonal variations affecting the distribution of dominant bacteria in boar semen, leading to the reduction of sperm quality and reproductive potential of boar. Optimal reproductive performance and effective bacterial control strategies are necessary to minimize the risk of bacterial contamination, including monitoring programs designed for quick detection and intervention. Therefore, further studies are needed to investigate the symbiotic relationships between sperm and semen microbial communities, for example, how the microorganisms that live with semen contribute to spermatozoa wellbeing and influence sperm fertilizing capacity. We need a better understanding of why bacteria differ between seasons and how these differences affect sperm biology.

## Conclusion

In conclusion, our studies showed that the boar semen microbiota of summer differed from that of winter semen, potentially due to seasonal changes related to semen quality and sperm fertilizing capacity. Higher bacterial diversity of ejaculated semen was observed in winter than in summer. Our results strongly indicated that *Lactobacillus* is not only a potential probiotic for semen quality and fertility potential, but also may be beneficial in restraining the negative influence of *Pseudomonas*. Overall, we provide data for a likely contribution to understand the environmental factors influencing the semen microbiota, and our findings significantly contribute to the current understanding of the phenotypes and etiology of male “summer infertility,” which may provide a frontier in male reproductive disorders and possible early prevention of pathogenic bacteria.

## Data Availability Statement

The datasets generated for this study can be found in the NCBI, No. PRJNA642992.

## Ethics Statement

The animal study was reviewed and approved by the Animal Ethics Committee of Shanghai Jiao Tong University.

## Author Contributions

XL designed, coordinated the study, and revised the manuscript. JZ performed on data analysis and drafted the manuscript. HL, QY, and PL performed the samples collection and DNA extraction. YW performed laboratory work. XH, BL, and HJ performed and advised on data analysis. All authors contributed to editing the manuscript and approved the final version.

## Conflict of Interest

The authors declare that the research was conducted in the absence of any commercial or financial relationships that could be construed as a potential conflict of interest.
