# Supplementary material for: Genomic Sequencing Reveals the Diversity of Seminal Bacteria and Relationships to Reproductive Potential in Boar Sperm
Source: Front Microbiol. 2020 Aug 4;11:1873. doi: 10.3389/fmicb.2020.01873 (PMC7438901; doi:10.3389/fmicb.2020.01873)
Supplement: Supplementary file 1 [file Data_Sheet_1.PDF]

Supplementary Materialc

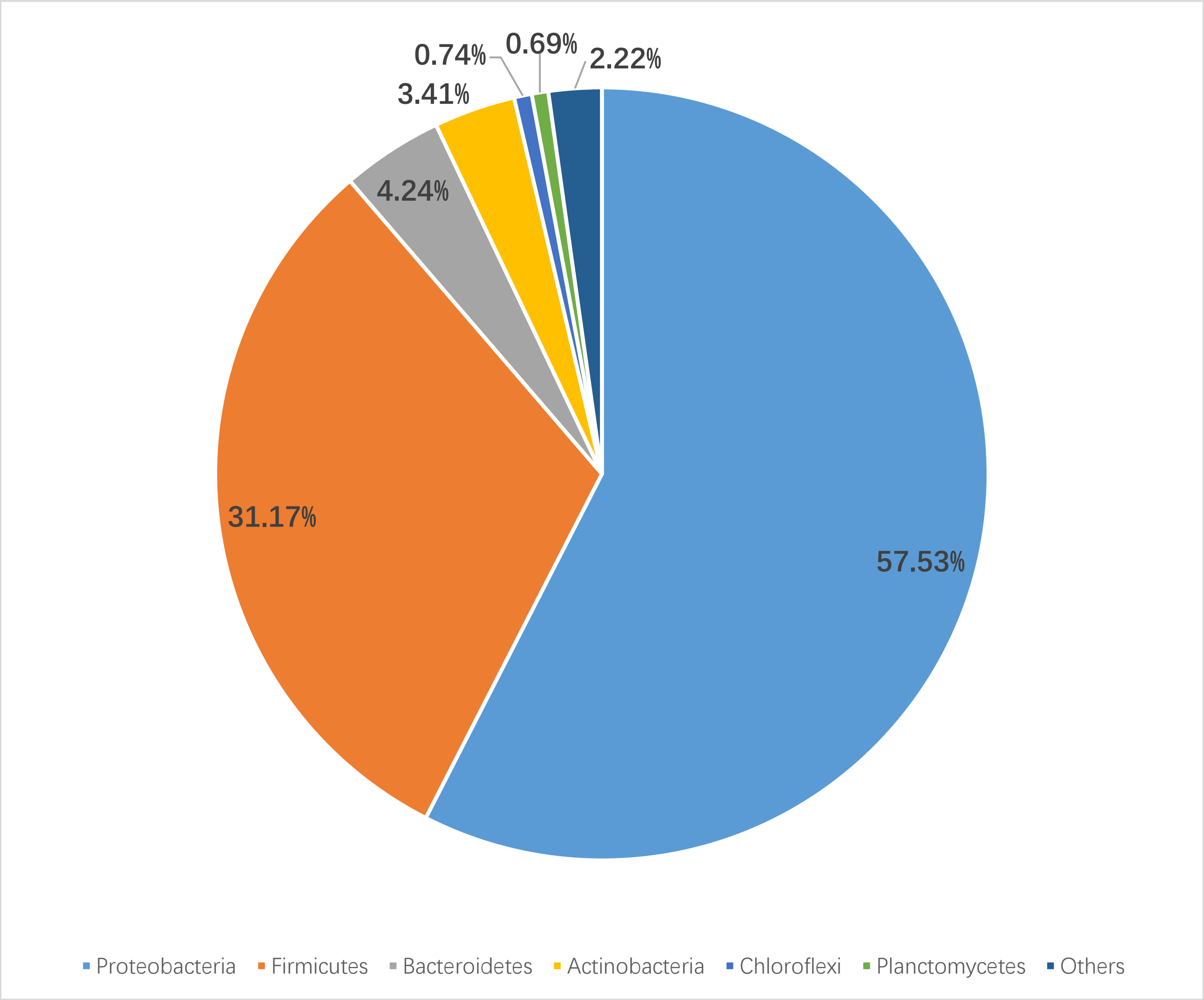

Supplementary Figure 1. Average relative abundance of dominant phyla in semen

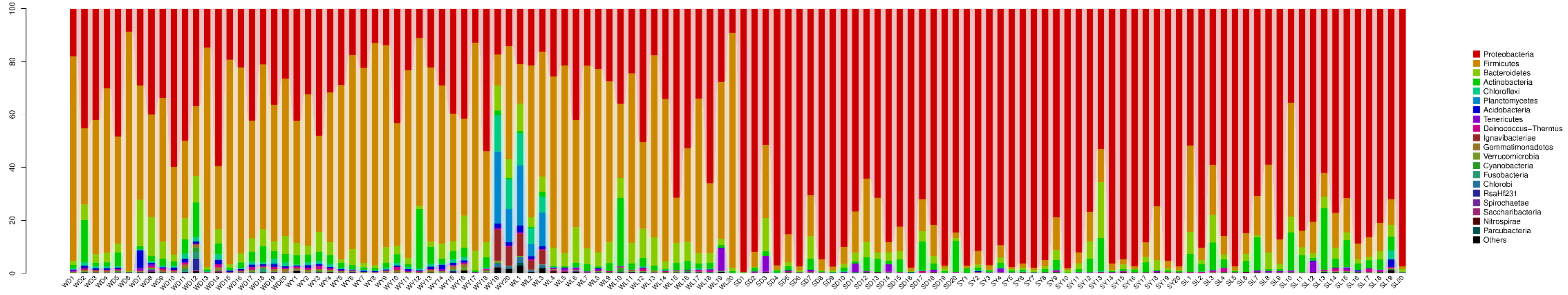

Supplementary Figure 2. Bacterial composition at phylum level in all semen samples. WD, samples collected from Duroc in winter; WY, samples collected from Yorkshire in winter; WL, samples collected from Landrace in winter; SD, samples collected from Duroc in summer; SY, samples collected from Yorkshire in summer; SL, samples collected from Landrace in summer. Numbers following WD, WY, WL, SD, SY, or SL indicate sample (boar semen) number.

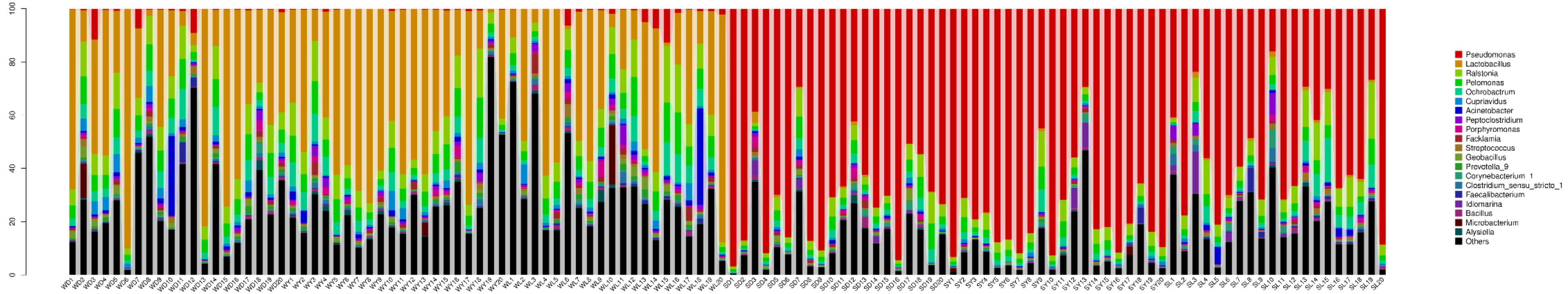

Supplementary Figure 3. Bacterial composition at genus level in all semen samples. WD, samples collected from Duroc in winter; WY, samples collected from Yorkshire in winter; WL, samples collected from Landrace in winter; SD, samples collected from Duroc in summer; SY, samples collected from Yorkshire in summer; SL, samples collected from Landrace in summer. Numbers following WD, WY, WL, SD, SY, or SL indicate sample (boar semen) number.

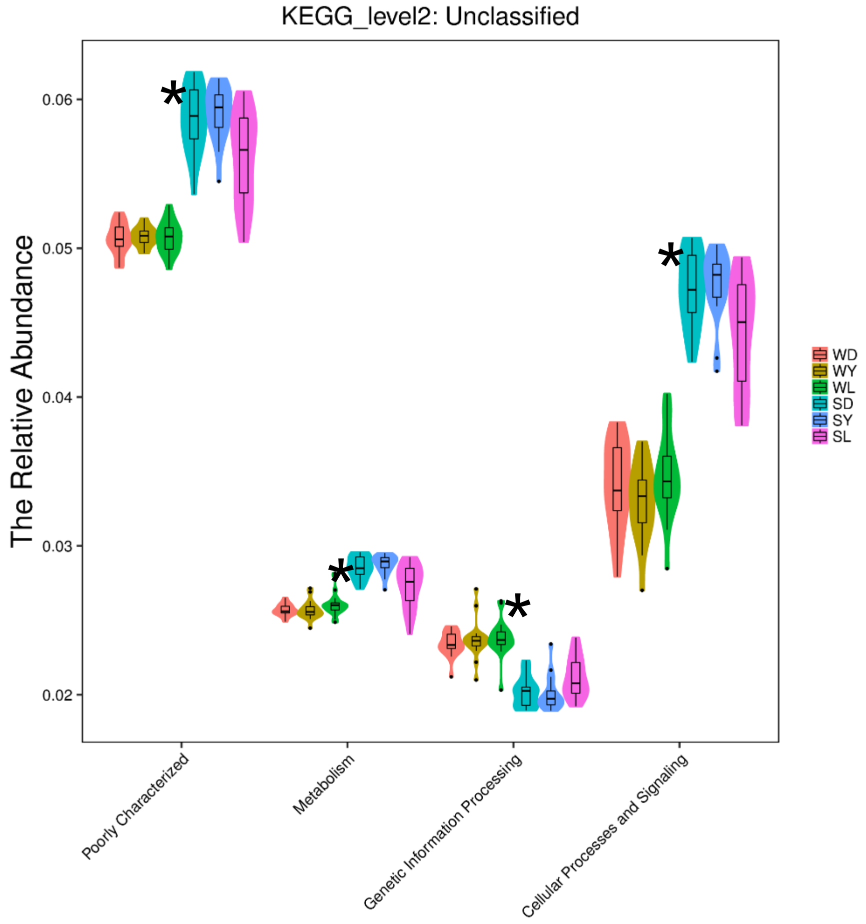

Supplementary Figure 4. Function prediction of semen bacteria in winter and summer – the unclassified second level of functional categories of the KEGG pathway. SD, samples collected from Duroc in summer; SL, samples collected from Landrace in summer; SY, samples collected from Yorkshire in summer; WD, samples collected from Duroc in winter; WL, samples collected from Landrace in winter ; WY, samples collected from Yorkshire in winter. The * means significant differences ($P < 0.05$) between summer and winter semen in all breeds of boars, and P value corrected for multiple testing according to the procedure of Benjamini-Hochberg.

Supplementary Table 1 Function prediction of semen bacteria - different abundance at second level of functional categories of the KEGG pathway between winter and summer

KEGG pathway	Duroc				Landrace				Yorkshire			
	C.P[1]	Winter ARC[2](%)	Summer ARC[2] (%)	Effect [3]	C.P[1]	Winter ARC[2] (%)	Summer ARC[2] (%)	Effect [3]	C.P[1]	Winter ARC[2] (%)	Summer ARC[2] (%)	Effect [3]
Cellular Processes; Cell Growth and Death	<0.01	0.49±0.02	0.34±0.03	-0.15	<0.01	0.48±0.02	0.38±0.05	-0.10	<0.01	0.49±0.02	0.34±0.03	-0.15
Cellular Processes; Cell Motility	<0.01	2.39±0.49	3.71±0.23	1.32	<0.01	2.55±0.63	3.41±0.33	0.86	<0.01	2.34±0.62	3.72±0.28	1.38
Environmental Information Processing; Membrane Transport	<0.01	15.06±1.27	14.02±0.28	-1.04	0.01	14.77±1.76	14.11±0.57	-0.66	<0.01	15.07±1.69	14.04±0.49	-1.03
Environmental Information Processing; Signaling Molecules and Interaction	<0.01	0.19±0.02	0.23±0.02	0.04	<0.01	0.17±0.03	0.21±0.02	0.04	<0.01	0.19±0.03	0.24±0.02	0.05
Environmental Information Processing; Signal Transduction	<0.01	2.03±0.16	3.03±0.21	1.00	<0.01	2.05±0.17	2.74±0.31	0.69	<0.01	1.97±0.16	3.06±0.23	1.09
Genetic Information Processing; Folding, Sorting and Degradation	<0.01	2.14±0.07	2.03±0.06	-0.11	<0.01	2.21±0.16	2.09±0.08	-0.12	<0.01	2.19±0.18	2.02±0.07	-0.17
Genetic Information Processing; Replication and Repair	<0.01	7.30±0.42	5.67±0.40	-1.63	<0.01	7.37±0.42	6.12±0.59	-1.25	<0.01	7.41±0.40	5.59±0.43	-1.82
Genetic Information Processing; Transcription	<0.01	2.46±0.07	2.60±0.07	0.14	<0.01	2.43±0.13	2.59±0.08	0.16	<0.01	2.43±0.14	2.62±0.06	0.19
Genetic Information Processing; Translation	<0.01	4.45±0.31	3.49±0.25	-0.96	<0.01	4.54±0.32	3.75±0.37	-0.79	<0.01	4.54±0.30	3.44±0.27	-1.10
Human Diseases; Cardiovascular Diseases	<0.01	0.01±0.00	0±0	-0.01	0.02	0.01±0.00	0±0	-0.01	<0.01	0.01±0.00	0±0	-0.01
Human Diseases; Infectious Diseases	<0.01	0.41±0.02	0.56±0.04	0.15	<0.01	0.41±0.02	0.51±0.05	0.10	<0.01	0.41±0.02	0.56±0.04	0.15
Human Diseases; Metabolic Diseases	<0.01	0.08±0.01	0.06±0.00	-0.02	<0.01	0.08±0.02	0.07±0.01	-0.01	<0.01	0.08±0.02	0.06±0.01	-0.02
Human Diseases; Neurodegenerative Diseases	<0.01	0.27±0.04	0.31±0.02	0.04	<0.01	0.26±0.05	0.31±0.03	0.05	<0.01	0.26±0.05	0.31±0.02	0.05
Metabolism; Amino Acid Metabolism	<0.01	9.73±0.60	10.73±0.13	1.00	<0.01	9.66±0.59	10.69±0.15	1.03	<0.01	9.61±0.45	10.78±0.11	1.17
Metabolism; Biosynthesis of Other Secondary Metabolites	<0.01	0.81±0.05	0.68±0.03	-0.13	<0.01	0.81±0.11	0.73±0.07	-0.08	<0.01	0.83±0.10	0.68±0.03	-0.15
Metabolism; Carbohydrate Metabolism	<0.01	10.85±0.76	8.70±0.27	-2.15	<0.01	10.74±0.78	9.05±0.44	-1.69	<0.01	11.01±0.69	8.67±0.24	-2.34
Metabolism; Energy Metabolism	<0.01	5.17±0.22	4.73±0.17	-0.44	<0.01	5.37±0.63	4.86±0.21	-0.51	<0.01	5.35±0.70	4.69±0.16	-0.66
Metabolism; Enzyme Families	<0.01	1.89±0.05	1.83±0.03	-0.06	<0.01	1.90±0.06	1.84±0.05	-0.06	<0.01	1.91±0.06	1.83±0.03	-0.08
Metabolism; Lipid Metabolism	<0.01	3.54±0.17	4.08±0.13	0.54	<0.01	3.47±0.18	3.94±0.19	0.47	<0.01	3.49±0.16	4.09±0.11	0.60
Metabolism; Metabolism of Other Amino Acids	<0.01	1.89±0.09	2.08±0.04	0.19	<0.01	1.82±0.13	2.03±0.08	0.21	<0.01	1.84±0.14	2.07±0.04	0.23
Metabolism; Metabolism of Terpenoids and Polyketides	<0.01	1.99±0.09	2.19±0.05	0.20	<0.01	1.95±0.08	2.14±0.07	0.19	<0.01	1.96±0.10	2.20±0.04	0.24
Metabolism; Nucleotide Metabolism	<0.01	3.39±0.25	2.60±0.18	-0.79	<0.01	3.42±0.25	2.80±0.26	-0.62	<0.01	3.46±0.23	2.57±0.19	-0.89

Metabolism; Xenobiotics Biodegradation and Metabolism	<0.01	3.58±0.45	4.02±0.17	0.44	<0.01	3.34±0.55	3.87±0.29	0.53	<0.01	3.34±0.55	4.02±0.16	0.68
Organismal Systems; Circulatory System	<0.01	0.04±0.01	0.06±0.00	0.02	<0.01	0.04±0.01	0.05±0.01	0.01	<0.01	0.04±0.01	0.06±0.00	0.02
Organismal Systems; Nervous System	<0.01	0.08±0.01	0.14±0.02	0.06	<0.01	0.08±0.01	0.13±0.02	0.05	<0.01	0.08±0.01	0.15±0.01	0.07
Unclassified; Cellular Processes and Signaling	<0.01	3.41±0.29	4.73±0.25	1.32	<0.01	3.47±0.28	4.43±0.38	0.96	<0.01	3.30±0.25	4.76±0.22	1.46
Unclassified; Genetic Information Processing	<0.01	2.34±0.08	2.02±0.10	-0.32	<0.01	2.38±0.12	2.12±0.13	-0.26	<0.01	2.37±0.12	2.00±0.11	-0.37
Unclassified; Metabolism	<0.01	2.57±0.04	2.86±0.07	0.29	0.01	2.61±0.08	2.73±0.14	0.12	<0.01	2.57±0.06	2.88±0.06	0.31
Unclassified; Poorly Characterized	<0.01	5.06±0.10	5.87±0.22	0.81	<0.01	5.07±0.10	5.61±0.30	0.54	<0.01	5.08±0.06	5.91±0.18	0.83

[1]C.P: P value corrected for multiple testing according to the procedure of Benjamini-Hochberg. This table only showed the KEGG pathways with C.P lower than 0.05 in all breeds of boars.

[2]ARC: Average relative contribution [%] of a KEGG pathway. Values represent means ± SDs.

[3] Effect: indicates whether the average relative contribution of a KEGG pathway was increased (positive number) or decreased (negative number) in summer comparing to winter.
